# Empowering Women’s Health: Insights Into HPV Vaccination and the Prevention of Invasive Cervical Cancer

**DOI:** 10.7759/cureus.49523

**Published:** 2023-11-27

**Authors:** Sachin Rathod, Jyotsna Potdar, Aishwarya Gupta, Neha Sethi, Anubha Dande

**Affiliations:** 1 Obstetrics and Gynecology, Jawaharlal Nehru Medical College, Datta Meghe Institute of Higher Education and Research, Wardha, IND

**Keywords:** public health interventions, global health initiatives, vaccine hesitancy, women's health, cervical cancer prevention, hpv vaccination

## Abstract

This review article provides a comprehensive analysis of the role of human papillomavirus (HPV) vaccination in empowering women's health by preventing invasive cervical cancer. Cervical cancer remains a significant global health concern, with HPV infection identified as a primary causative factor. The review synthesizes current research findings, epidemiological data, and clinical outcomes to offer a nuanced understanding of the impact of HPV vaccination on cervical cancer prevention. The article explores the biology of HPV, emphasizing its association with cervical carcinogenesis and the development of precancerous lesions. It delves into the epidemiology of HPV infection, highlighting the global prevalence and the burden of cervical cancer, particularly in low-resource settings. The socio-economic factors influencing HPV transmission and cervical cancer incidence are also discussed. The focus of the review is on the efficacy and safety of HPV vaccines, including an examination of vaccine types, dosages, and long-term effectiveness. Insights into the immunological responses triggered by vaccination, as well as the duration of protection against HPV infection and associated cancers, are presented. Special attention is given to addressing myths and misconceptions surrounding HPV vaccination, aiming to enhance public awareness and acceptance. Additionally, the review discusses the impact of vaccination programs on population-wide HPV prevalence and the subsequent reduction in cervical cancer rates. It explores challenges related to vaccine accessibility, affordability, and coverage, especially in underserved populations. Strategies for improving vaccine uptake and ensuring equitable distribution are considered, with a focus on empowering women through education and healthcare access. Furthermore, the article touches upon emerging trends in HPV research, such as the development of next-generation vaccines, therapeutic interventions for existing infections, and the potential for global HPV elimination. The integration of HPV vaccination into comprehensive women's health programs is advocated, emphasizing the importance of a holistic approach to cervical cancer prevention. In conclusion, this review provides a multifaceted exploration of HPV vaccination as a pivotal tool in empowering women's health by preventing invasive cervical cancer. The synthesis of current knowledge underscores the need for continued research, education, and global collaboration to ensure the widespread success of HPV vaccination initiatives, ultimately contributing to the advancement of women's health worldwide.

## Introduction and background

Women's health encompasses a broad spectrum of physical, mental, and social well-being, making it a critical aspect of overall societal prosperity. Throughout history, addressing women's health has been paramount for achieving gender equity and promoting the well-being of communities. Understanding the unique healthcare needs of women is crucial for fostering a healthier and more equitable society [1].

Among the myriad health concerns affecting women, human papillomavirus (HPV) and its link to cervical cancer stand out as a pressing issue. Cervical cancer is a significant health issue in India, with the disease being the leading cancer in Indian women and the second most common cancer in women worldwide. The implications of cervical cancer extend beyond individual health, affecting families, communities, and healthcare systems [2]. Cervical cancer is often preventable, and understanding the significance of addressing HPV is pivotal in reducing the burden of this disease. Through proactive measures such as vaccination, early detection, and education, it is possible to mitigate the impact of HPV on women's health and prevent the occurrence of invasive cervical cancer [3].

The purpose of this article is to provide comprehensive insights into the role of HPV vaccination in empowering women's health and preventing invasive cervical cancer. By delving into the various facets of HPV, cervical cancer, and vaccination, this article aims to contribute to the collective knowledge surrounding women's health issues. Additionally, the article seeks to highlight the importance of raising awareness, addressing challenges, and promoting effective strategies to enhance the uptake of HPV vaccines, ultimately striving toward the goal of eliminating cervical cancer as a public health concern.

## Review

Cervical cancer: a global perspective

Incidence and Prevalence of Cervical Cancer Worldwide

In 2020, there were an estimated 604,127 cervical cancer cases and 341,831 deaths globally, with an age-standardized incidence of 13.3 cases per 100,000 women-years and a mortality rate of 7.2 deaths per 100,000 women-years [4]. The incidence of cervical cancer ranged from 2.2 in Iraq to 84.6 in Eswatini, while mortality rates ranged from 1.0 in Switzerland to 55.7 in Eswatini [4]. The burden of cervical cancer is higher in low- and middle-income countries (LMICs), with about 90% of deaths occurring in these regions [5]. The high mortality rate from cervical cancer globally could be reduced by effective interventions at different stages of life, including HPV vaccination and screening programs [5]. It is important to continue tracking changing trends in cervical cancer epidemiology to inform healthcare strategies and work toward the global elimination of cervical cancer as a public health problem [6].

Disparities in Cervical Cancer Rates Among Different Populations

Rural-urban disparities: A study by the Centers for Disease Control and Prevention (CDC) found that the effect of rurality varied by race/ethnicity. White women had a higher incidence of cervical cancer in rural counties for any stage. However, Hispanic and non-Hispanic black women had a higher incidence of regional and distant cervical cancer than non-Hispanic white women. According to estimates, approximately 132,000 new cases of cervical cancer are diagnosed annually in India, accounting for nearly one-third of global cervical cancer deaths [7]. This highlights the need for continued efforts to provide and promote cervical cancer screening in rural areas and among minority women [7].

Screening disparities: Another study revealed racial and ethnic disparities in cervical cancer screening across diverse healthcare settings in the U.S. It found that screening use was lower among non-Hispanic Black patients and higher among Hispanic and Asian/Pacific Islander patients compared to non-Hispanic White patients. The distribution of patients across sites and differences in insurance partly explained the differences. The study emphasized the role of systemic inequity in contributing to disparities in screening and follow-up after abnormalities are identified [8].

Incidence disparities by histologic subtype: Research published in ASCO Journals indicated that incidence rates of cervical squamous cell carcinoma were highest in Black and Hispanic women. In contrast, incidence rates of cervical adenocarcinoma were highest in Asian/Pacific Islander women. This suggests variations in the incidence of different histologic subtypes of cervical cancer among different racial and ethnic groups [9]. These findings underscore the importance of addressing disparities in cervical cancer rates among different populations. Efforts to improve access to screening, vaccination, and follow-up care, particularly in rural and minority communities, are crucial in reducing the burden of cervical cancer and promoting women's health.

Impact on Women's Health and Society

Women's health significantly impacts society, and improving women's health outcomes can have far-reaching benefits. Studies show healthier women and their children contribute to more productive and better-educated communities [10]. Women are the bedrock of our economy and communities, and prioritizing women's health can correct the historical underrepresentation of women across industries and accelerate future economic growth and health equity progress [11]. However, there are significant gaps and disparities in women's health, particularly among minority and rural populations [12]. Poverty, social exclusion, unemployment, poor working conditions, and unequal gender relations are some of the factors that have a profound influence on patterns of women's health [13]. Therefore, addressing systemic inequities and promoting access to quality health services and education is crucial in improving women's health and promoting gender equity.

The role of HPV vaccination in preventing cervical cancer

Mechanism of Action of HPV Vaccines

To comprehend the preventive power of HPV vaccines, it is essential to delve into their mechanism of action. HPV vaccines stimulate the immune system to produce an immune response against specific HPV types. Most HPV vaccines target the high-risk types, such as HPV-16 and HPV-18, which are known to cause most cervical cancer cases. The vaccines typically contain virus-like particles that resemble HPV but do not contain the genetic material necessary for viral replication. When administered, these particles prompt the immune system to produce antibodies, protecting against future infections [14]. Understanding the intricacies of how HPV vaccines induce immunity is crucial for appreciating their role in cervical cancer prevention and underscores the significance of vaccination as a primary preventive measure [3].

Efficacy in Preventing HPV Infection and Related Cancers

Efficacy against cervical cancer: Clinical trials and large-scale studies have consistently shown that HPV vaccines are highly effective in preventing cervical infection with the types of HPV they target, particularly HPV serotypes 16 and 18, which are responsible for approximately 70% of cervical cancer cases [14-16]. A large study in Sweden confirmed that widespread use of the HPV vaccine dramatically reduces the number of women who will develop cervical cancer, with a nearly 90% reduction in cervical cancer incidence among girls vaccinated before age 17 [15]. The efficacy and effectiveness of the quadrivalent HPV vaccine in preventing high-grade cervical lesions have been well-documented, further supporting its role in preventing invasive cervical cancer [17].

Prevention of other HPV-related cancers: HPV vaccines have also been found to reduce infections in other tissues that HPV infects, including the anus and oral region, indicating a broader protective effect against HPV-related cancers beyond cervical cancer [14]. Clinical trials and real-world data have demonstrated that the vaccines greatly reduce the risk of precancers and cancers of the cervix, vagina, and vulva in vaccinated women [18]. Additionally, HPV vaccination can prevent 33,700 cancers by preventing the infections that cause them, including cancers of the anus, penis, vagina, vulva, and the back of the throat (oropharyngeal cancer). The HPV vaccine provides safe, effective, and lasting protection against the HPV infections that most commonly cause cancer [18].

Public health impact: Widespread HPV vaccination has the potential to reduce cervical cancer incidence around the world by as much as 90%, highlighting the significant public health impact of HPV vaccination in preventing cervical cancer [14]. The combination of HPV vaccination and cervical screening can provide the greatest protection against cervical cancer and may reduce the need for subsequent medical care, biopsies, and invasive procedures associated with abnormal cervical screening, thereby reducing healthcare costs and anxieties related to follow-up procedures [14]. These findings collectively emphasize the substantial efficacy of HPV vaccination in preventing HPV infection and related cancers, particularly cervical cancer, and underscore its potential to have a significant impact on global public health.

Importance of Vaccination in Reducing Cervical Cancer Rates

Potential for cervical cancer elimination: Comparative modeling analysis in LMICs suggests that girls-only HPV vaccination could lead to cervical cancer elimination in 99% of LMICs, based on a threshold of ten years [19]. Statistical models indicate the possibility of eliminating cervical cancer within the next century, contingent upon achieving vaccination rates of 80% [20].

Global impact and disproportionate burden: HPV vaccination represents a crucial opportunity to significantly reduce the global burden of cervical cancer, particularly in developing countries where over 80% of cervical cancer cases occur [21]. The burden of preventable cervical cancer disproportionately affects women in countries with weak or non-existent cervical cancer screening and treatment systems, highlighting the critical role of vaccination in these settings [21].

Efficacy and effectiveness: Widespread use of the HPV vaccine has been shown to dramatically reduce the incidence of cervical cancer, with a nearly 90% reduction in cervical cancer incidence among girls vaccinated before age 17 [15]. Studies have demonstrated the high efficacy of HPV vaccination in protecting young women against cervical infection with cancer-causing HPV types, with a single dose providing 97.5% protection against new persistent infections with HPV 16 and 18 [22]. These findings collectively emphasize the pivotal role of HPV vaccination in reducing cervical cancer rates, with the potential to eliminate cervical cancer in many regions. The evidence underscores the importance of achieving high vaccination coverage, particularly in low-resource settings, to effectively combat the burden of cervical cancer and protect women's health.

Challenges and barriers to HPV vaccination

Vaccine Hesitancy and Misinformation

Despite the proven efficacy and safety of HPV vaccines, one of the foremost challenges in their widespread adoption is vaccine hesitancy fueled by misinformation. Myths and misconceptions surrounding the safety and purpose of HPV vaccination contribute to a reluctance among individuals and parents to seek vaccination for themselves or their children. Addressing these misconceptions through targeted education campaigns is imperative to dispel fears and foster confidence in the benefits of HPV vaccination [23]. In the era of rapid information dissemination through various media channels, countering misinformation requires a concerted effort from healthcare professionals, public health campaigns, and community leaders. Providing accurate information and addressing concerns can overcome vaccine hesitancy and encourage greater acceptance of HPV vaccination as a vital preventive measure [24].

Access to Vaccination Services

Limited access to vaccination services poses a significant barrier to achieving widespread HPV vaccine coverage. Disparities in healthcare infrastructure, particularly in low-income and rural areas, can hinder individuals' ability to receive timely and affordable vaccinations. Improving access involves addressing logistical challenges, ensuring the availability of vaccines in diverse settings, and implementing outreach programs to reach underserved populations [25]. Efforts to expand access must consider the socio-economic factors that contribute to disparities. This includes promoting vaccination in schools, community centers, and healthcare facilities and implementing mobile vaccination clinics to reach remote areas. Addressing access issues is integral to maximizing the impact of HPV vaccination on a global scale [26].

Cultural and Societal Factors Influencing Vaccine Uptake

Cultural and societal factors play a pivotal role in shaping attitudes toward vaccination. Prevailing beliefs, traditions, and societal norms can influence the acceptance or rejection of vaccines, including HPV vaccines. Sensitivity to cultural contexts is essential for designing effective vaccination strategies that resonate with diverse communities [27]. Understanding cultural perceptions and involving community leaders and influencers in vaccination campaigns can help bridge gaps and foster acceptance. Tailoring educational materials to address cultural concerns and highlighting the importance of vaccination in the context of community well-being can contribute to overcoming cultural and societal barriers to HPV vaccine uptake [28].

Promoting HPV vaccination: strategies and interventions

Education and Awareness Campaigns

Effective education and awareness campaigns are instrumental in dispelling myths, combating misinformation, and promoting the benefits of HPV vaccination. Targeted campaigns should focus on providing accurate information about the safety and efficacy of the vaccines, addressing common concerns, and emphasizing the long-term impact of vaccination on preventing cervical cancer. Utilizing various channels such as social media, community workshops, and healthcare provider interactions can amplify the reach of these campaigns, ensuring that accurate information reaches diverse audiences [29]. Incorporating education on HPV vaccination into school curricula and partnering with healthcare professionals to serve as community advocates can contribute to a well-informed public, fostering a positive attitude toward vaccination [30].

Improving Access to Vaccination Services

Enhancing access to vaccination services is crucial for reaching underserved populations. This involves implementing practical solutions such as expanding vaccination programs to schools, workplaces, and community centers and establishing mobile vaccination clinics to reach remote areas. Collaboration between public health agencies, healthcare providers, and community organizations can facilitate the development of targeted initiatives to ensure vaccines are readily available and accessible [31]. Reducing financial barriers through subsidies or free vaccination programs and addressing logistical challenges, such as vaccine storage and transportation, are key components of improving access to HPV vaccination services [32].

Addressing Cultural and Societal Barriers

Acknowledging and understanding cultural and societal factors influencing vaccine uptake is essential for tailoring interventions. Community engagement initiatives involving local leaders and influencers can help build trust and overcome cultural barriers. Developing culturally sensitive educational materials and campaigns that resonate with diverse communities can change perceptions and foster a positive attitude toward vaccination [33,34]. Collaboration with community-based organizations and leveraging existing cultural networks can provide valuable insights and aid in designing interventions that align with the beliefs and values of specific populations [1]. In the case of HPV vaccination in India, community-based organizations such as CAPED have been eager to kickstart outreach programs, waiting for months to receive official communication guidelines from the health ministry [34]. Authorities have also held workshops on the vaccine for local media, which is crucial due to previous negative coverage of the HPV vaccine [14]. In addition to community-based organizations, healthcare providers and public health officials can play a critical role in promoting HPV vaccination and addressing vaccine hesitancy. The CDC recommends that healthcare providers strongly recommend HPV vaccination to their patients and their parents [14]. The CDC also recommends that public health officials work with healthcare providers, schools, and community organizations to increase HPV vaccination rates [14].

Policy Recommendations and Advocacy

Policy plays a pivotal role in shaping the landscape of vaccination. Advocacy efforts should aim to influence local, national, and international policy decisions. This includes advocating for the inclusion of HPV vaccination in national immunization programs, ensuring coverage under healthcare plans, and implementing policies that support school-based vaccination programs [35]. Engaging with policymakers, healthcare professionals, and advocacy groups can amplify the voice for HPV vaccination, promote policies that enhance vaccine access, reduce financial barriers, and foster a supportive environment for vaccination efforts [36]. By implementing a multifaceted approach that combines education, improved access, cultural sensitivity, and policy advocacy, it is possible to promote HPV vaccination effectively, ultimately reducing the burden of cervical cancer and advancing women's health on a global scale [37].

Success stories and best practices

Examples of Countries or Regions With Successful HPV Vaccination Programs

Geneva Canton, Switzerland: In Geneva Canton, Switzerland, the implementation of the HPV vaccination program achieved remarkable success within a short period. The immunization coverage reached an impressive 82% of the primary target population. This high coverage rate suggests the program's effectiveness in reaching and vaccinating a significant proportion of the intended recipients. The success in Geneva may be attributed to efficient planning, robust healthcare infrastructure, and community engagement strategies that facilitated widespread awareness and acceptance of the HPV vaccine [38].

Rwanda: Rwanda's national HPV vaccination program stands out as another exemplary model of success, particularly due to its school-based rollout. The program attained an impressive 93% coverage among girls in grade six, indicating a high level of completion for the three-dose series. The achievement in Rwanda underscores the feasibility and effectiveness of integrating HPV vaccination into school health programs. The success is likely attributed to the collaboration between the health sector and educational institutions and the country's commitment to comprehensive healthcare initiatives [39].

Uzbekistan: Uzbekistan demonstrated significant success in achieving high HPV vaccination coverage against cervical cancer, with an outstanding 94% coverage rate among girls aged 12-14 for the first dose of the HPV vaccine. The success in Uzbekistan is linked to a multifaceted approach that includes educating key target groups, fostering collaboration among stakeholders, and promoting the broader societal benefits of protecting girls' health for future motherhood. This example highlights the importance of a holistic strategy beyond vaccination, emphasizing education and advocacy [40].

Low- and middle-income countries: A broader perspective on the success of HPV vaccination programs is evident in various LMICs. The study found that school-based models were effective in Peru, Uganda, Vietnam, and India demonstration programs. Furthermore, non-governmental organizations (NGOs) management appeared to be a predictive factor for vaccination coverage. These findings suggest successful HPV vaccination programs can be implemented in diverse settings and regions, showcasing adaptability [41]. The key success factors identified across these examples include effective education and awareness campaigns, strong stakeholder engagement involving governmental and non-governmental entities, and implementing innovative delivery models to overcome logistical challenges.

Lessons Learned and Replicable Strategies

Target groups and venues for vaccination: Delivering the HPV vaccine through easily accessible primary schools can achieve high coverage levels at reasonable incremental program costs. Early coordination between the health and education sectors is necessary to establish a feasible vaccination schedule for a multi-dose vaccine. Health workers should aim to visit schools just once per dose, and follow-up of girls who miss doses should be carried out through health centers [42].

Operational issues: Developing countries often face challenges when introducing new vaccines, including financing, health worker training, strengthening cold chain and storage capacity, and educating communities. Evaluation of vaccine coverage, acceptability, and feasibility of implemented strategies is crucial for assessing the program's success [42].

Communication and community engagement: Widespread social mobilization and education of healthcare workers, teachers, and community members can help maintain high coverage and build confidence in the vaccination program. Tailored strategies to introduce the HPV vaccine, including community engagement and cost consideration, can help build sustainable vaccination programs [43].

Lessons from low- and middle-income countries: Sensitization strategies about vaccination before the launch of vaccination campaigns are essential. Erroneous perceptions of the population related to the vaccine's safety and efficacy, as well as reaching and maintaining follow-up with target populations, are identified as challenges that need to be addressed. Coupling HPV vaccination with other health interventions for mothers of targeted girls helped to increase vaccination and cervical cancer screening [44].

Global health organization recommendations: The World Health Organization (WHO) recommends that routine HPV vaccination be included in national immunization programs based on public health priorities, program feasibility, sustainable financing, and cost-effectiveness. Effective education and mobilization of key stakeholders, including parents, health workers, and communities, are crucial for the success of HPV vaccination programs. These lessons and strategies highlight the importance of tailored approaches, community engagement, and effective coordination between health and education sectors in implementing successful HPV vaccination programs. They can serve as valuable guidance for countries and regions aiming to establish or improve their HPV vaccination initiatives [45].

Future directions and innovations

Ongoing Research and Development in HPV Vaccines

The landscape of HPV vaccination is continuously evolving through ongoing research and development efforts. Researchers are exploring new avenues to enhance vaccine efficacy, broaden the spectrum of protection, and improve the accessibility of HPV vaccines. Investigating the potential for cross-protection against additional HPV types and extending vaccine coverage to older age groups are areas of active exploration [46]. Furthermore, developing next-generation vaccines, including therapeutic vaccines for individuals already infected with HPV, represents a promising avenue in the quest for more comprehensive preventive measures. Tracking these advancements is critical for staying at the forefront of cervical cancer prevention and ensuring that new developments are translated into effective public health strategies [47].

Emerging Technologies and Interventions

Innovative technologies are pivotal in shaping the future of HPV prevention. Novel diagnostic tools for early detection of HPV infection and associated abnormalities are emerging, providing opportunities for more targeted and personalized interventions. Advancements in telemedicine and digital health platforms facilitate improved vaccine delivery systems, including remote monitoring and follow-up [48]. Additionally, integrating artificial intelligence (AI) and machine learning in cervical cancer screening and vaccination programs can enhance accuracy, streamline processes, and identify high-risk populations more effectively. Keeping abreast of these technological innovations is crucial for maximizing their impact on cervical cancer prevention and women's health [49].

Global Efforts Toward Cervical Cancer Elimination

The global community is increasingly recognizing the importance of collaborative efforts in the fight against cervical cancer. International organizations, governments, and non-governmental entities are working together to develop and implement strategies for cervical cancer elimination. These efforts include establishing vaccination programs, improving screening infrastructure, and fostering international partnerships to address disparities in access to preventive measures [50]. The WHO's global call for action toward the elimination of cervical cancer as a public health problem by the end of the century underscores the collective commitment to this cause. Monitoring and supporting these global initiatives are essential for ensuring that resources are effectively mobilized, policies are harmonized, and progress toward cervical cancer elimination is sustained [51].

## Conclusions

In conclusion, the comprehensive examination of HPV vaccination and its pivotal role in preventing invasive cervical cancer has brought to light crucial aspects of women's health on a global scale. Beginning with a recognition of the broader landscape of women's well-being, we delved into the mechanisms and efficacy of HPV vaccines. The exploration extended to a global perspective on cervical cancer, emphasizing disparities among diverse populations. Unveiling challenges such as vaccine hesitancy, limited access, and cultural influences, we proposed strategies ranging from education campaigns to policy initiatives. The call to action is resounding, demanding collaborative efforts to address these challenges. Success in promoting HPV vaccination not only prevents cervical cancer but also has a profound impact on women's health, reducing the emotional, physical, and economic burdens associated with the disease. The broader vision is one of societal commitment to equality, health, and the eradication of preventable diseases, ultimately empowering women to lead healthier, more fulfilling lives. As we look ahead, sustained efforts in research, education, and global collaboration are paramount to achieving a world where cervical cancer is rare, and women can thrive in optimal health and well-being. This collective journey toward a healthier future requires ongoing dedication and action from individuals, communities, and nations worldwide.
